# The 1:1 charge-transfer complex dibenzo­tetra­thia­fulvalene–pyromellitic dianhydride (DBTTF–PMDA)

**DOI:** 10.1107/S1600536814013324

**Published:** 2014-07-02

**Authors:** Margaret E. Payne, Katelyn P. Goetz, Cynthia S. Day, Oana D. Jurchescu

**Affiliations:** a Department of Physics, Wake Forest University, Winston-Salem, NC 27109, USA; b Department of Chemistry, Wake Forest University, Winston-Salem, NC 27109, USA

## Abstract

The title charge-transfer (CT) complex, C_10_H_2_O_6_·C_14_H_8_S_4_, composed of donor dibenzo­tetra­thia­fulvalene (DBTTF) and acceptor pyromellitic dianhydride (PMDA), forms a mixed stacking pattern along the [-110] direction. The constituent mol­ecules occupy crystallographic inversion centers. They are nearly parallel and lie *ca.*3.41 Å from each other. The crystals exhibit a high degree of donor/acceptor overlap [88.20 (4)%] in the long direction of the DBTTF and PMDA mol­ecules as compared with 51.27 (5)% in the shortest direction of the mol­ecules.

## Related literature   

General properties and potential applications of charge-transfer complexes in electronic devices are outlined by Goetz *et al.* (2014 ▶); Horiuchi *et al.* (2006 ▶); Tsutsumi *et al.* (2012 ▶); Kobayashi *et al.* (2012 ▶); Kagawa *et al.* (2010 ▶); Herbstein (2005 ▶); Ferraris *et al.* (1973 ▶); Kistenmacher *et al.* (1981 ▶); Takahashi *et al.* (2006 ▶); Wu *et al.* (2013 ▶). Related CT structures, containing the acceptor pyromellitic dianhydride (PMDA) include anthracene–PMDA (Robertson & Stezowski, 1978 ▶), phenanthrene–PMDA (Evans & Robinson, 1977 ▶), pyrene–PMDA (Herbstein & Snyman, 1969 ▶) and two polymorphs of bi­phenyl­ene–PMDA (Stezowski *et al.*, 1986 ▶). Structure–property relationships in mol­ecular crystals have been described theoretically by Coropceanu *et al.* (2007 ▶) and experimentally by Mei *et al.* (2013 ▶), among others.
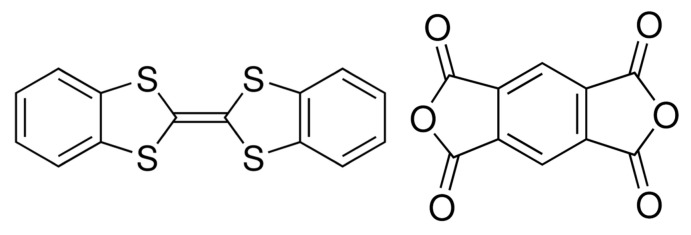


## Experimental   

### 

#### Crystal data  

C_10_H_2_O_6_·C_14_H_8_S_4_
*M* *_r_* = 522.56
Triclinic, 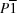

*a* = 7.2292 (4) Å
*b* = 8.9572 (5) Å
*c* = 9.5224 (5) Å
α = 70.051 (1)°
β = 68.712 (1)°
γ = 70.136 (1)°
*V* = 523.39 (5) Å^3^
*Z* = 1
Mo *K*α radiation
μ = 0.50 mm^−1^
*T* = 213 K
0.20 × 0.20 × 0.02 mm

#### Data collection  

Bruker APEX CCD diffractometer
Absorption correction: multi-scan (*SADABS*; Sheldrick, 2012 ▶) *T*_min_ = 0.703, *T*_max_ = 0.746
10004 measured reflections
3023 independent reflections
2668 reflections with *I* > 2σ(*I*)
*R*_int_ = 0.021

#### Refinement  

*R*[*F* ^2^ > 2σ(*F* ^2^)] = 0.032
*wR*(*F* ^2^) = 0.091
*S* = 1.07
3023 reflections
154 parameters
H-atom parameters constrained
Δρ_max_ = 0.30 e Å^−3^
Δρ_min_ = −0.33 e Å^−3^

### 

Data collection: *SMART* (Bruker, 2002 ▶); cell refinement: *SAINT* (Bruker, 2011 ▶); data reduction: *SAINT*; program(s) used to solve structure: SHELXLS2013 (Sheldrick, 2008 ▶); program(s) used to refine structure: *SHELXL2013* (Sheldrick, 2008 ▶); molecular graphics: *SHELXTL* (Sheldrick, 2008 ▶); software used to prepare material for publication: *SHELXTL*.

## Supplementary Material

Crystal structure: contains datablock(s) I. DOI: 10.1107/S1600536814013324/pk2526sup1.cif

Structure factors: contains datablock(s) I. DOI: 10.1107/S1600536814013324/pk2526Isup2.hkl

Supporting information file. DOI: 10.1107/S1600536814013324/pk2526Isup3.cml

CCDC reference: 1007162

Additional supporting information:  crystallographic information; 3D view; checkCIF report

## Figures and Tables

**Fig. 1.**
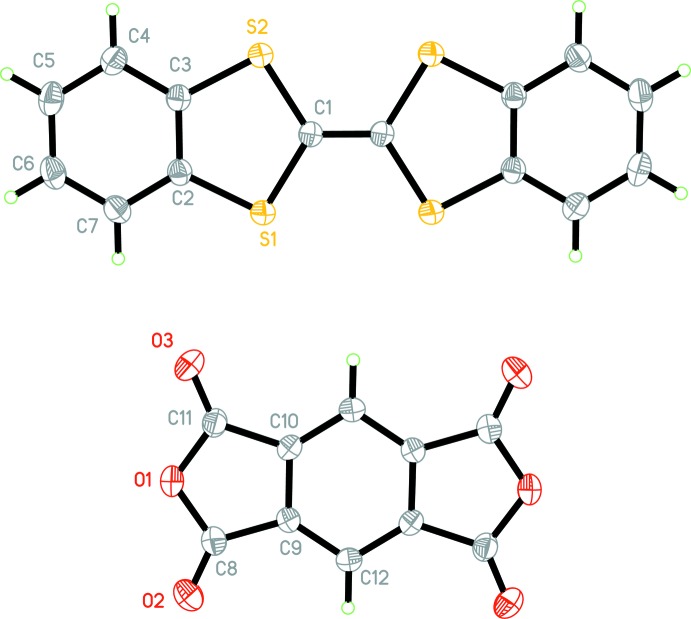
The atom numbering scheme for DBTTF-PMDA with non-hydrogen atoms represented by 50% probability ellipsoids. Each molecule occupies an inversion center in the unit cell and only contents of the asymmetric unit are labeled.

**Fig. 2.**
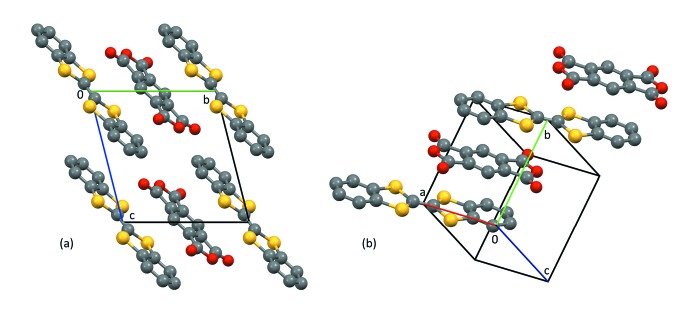
Molecular packing viewed down the a axis (a) and the view across a stack of donor and acceptor molecules (b) with the six-membered rings vertically aligned.

**Fig. 3.**
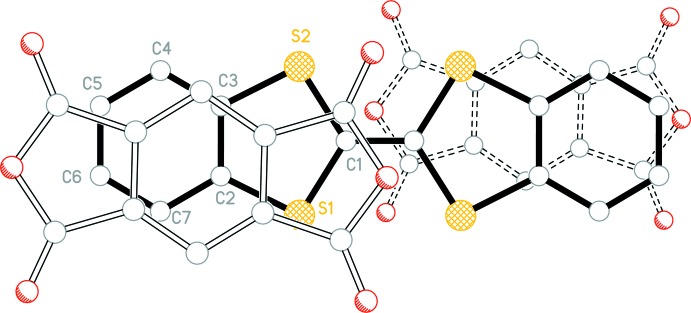
Projection perpendicular to plane of DBTTF with PMDA (above - open bonds) (below - dashed open bonds) showing overlap of rings.

## supplementary crystallographic information

### S1. Comment

Organic charge-transfer (CT) complexes are combinations of electron donor (D) and electron acceptor (A) materials. They have been studied for decades, but have attracted significant inter­est recently due to their intriguing properties such as photoconductivity, tunable semiconductivity, metallicity, ferroelectricity, *etc.*, which make them viable candidates for versatile electronic devices (Goetz *et al.*, 2014; Horiuchi *et al.*, 2006; Tsutsumi *et al.*, 2012; Kobayashi *et al.*, 2012; Kagawa *et al.*, 2010). In the 1:1 D:A stoichiometry, they can exhibit either mixed stacking, where the repeating motif in the π-stacking direction is ···D—A—D—A···, or segregated stacking, where the donor and acceptor π-stack separately, as ···A—A—A—A··· and ···D—D—D—D··· (Herbstein, 2005). CT complexes of the acceptor 7,7,8,8-tetra­cyano­quinodi­methane (TCNQ) have been widely explored. Examples include the organic metal with donor tetra­thia­fulvalene (TTF) (Ferraris *et al.*, 1973) or the ambipolar semiconductor with dibenzo­tetra­thia­fulvalene (DBTTF) (Kistenmacher *et al.*, 1981; Takahashi *et al.*, 2006; Wu *et al.*, 2013). With the exception of a few reports, CT complexes containing pyromellitic dianhydride (PMDA) as an acceptor have received little attention. Examples of CT complexes of PMDA include anthracene-PMDA (Robertson & Stezowski, 1978), phenanthrene-PMDA (Evans & Robinson, 1977), pyrene-PMDA (Herbstein & Snyman, 1969), and two polymorphs of bi­phenyl­ene-PMDA (Stezowski *et al.*, 1986). Here we report for the first time on the growth and crystal structure of the 1:1 CT complex containing the donor DBTTF and acceptor PMDA.

Single crystals of DBTTF-PMDA are triclinic, space group P1, with Z=1, where the DBTTF and PMDA molecules occupy crystallographic inversion centers at (0,0,0) and (1/2,1/2,0), respectively. The crystals are platelets, with their largest face corresponding to the (001) plane. The molecular structure is shown with thermal ellipsoids in Figure 1, where only the contents of the asymmetric unit are labeled. The DBTTF and PMDA molecules pack in a mixed-stack pattern, previously observed in other PMDA-based CT complexes. As shown in Figure 2, the DBTTF molecules lie on the corners of the unit cell, while the PMDA molecules lie in the center of the *ab* crystal faces. The mixed DA stacks build along the [-1 1 0] direction and are tilted by 45.43 (6)° (DBTTF) and 46.40 (6)° (PMDA) with respect to *ab* face. This tilt leads to a molecular overlap between the donor and acceptor wherein the fused 5-and 6-membered rings of each half DBTTF overlap ("straddle") the 3 fused rings of the PMDA molecules in the stack (Figure 3). The centroid of the central PMDA 6-membered ring to centroid of the 5- and 6-membered rings of the adjacent DBTTF molecules in the stack are 3.648 (1)Å and 3.585 (1)Å, respectively. The shortest centroid-centroid contact involving 5-membered rings of the DBTTF and PMDA molecules is 3.611 (1)Å while the shortest contacts involving the DBTTF 6-membered ring centroid are 3.527 (1)Å and 3.538 (1)Å to the centroids of the 5-membered PMDA and 6-membered DBTTF rings, respectively. The planes of the D/A molecules are nearly parallel with an inter­planar angle of 1.31 (5)° and the long axes of the DBTTF and PMDA are also nearly parallel [1.8 (1)°]. The PMDA and DBTTF are symmetrically-spaced within each stack with an inter­molecular separation of 3.408 (1)Å. This differs from anthracene-PMDA, where the DA spacing alternates between 3.32Å and 3.4Å (Robertson & Stezowski, 1978). There is a high degree of overlap between donor and acceptor molecules: the DBTTF molecule overlaps with 88.20 (4)% of the PMDA molecule in the longest direction of the molecule, and with 51.27 (5)% of the PMDA molecule in the shortest direction of the molecule. Measurements are in progress to evaluate the degree of charge transfer between the two moieties; however, this high degree of overlap suggests that a high value is to be expected. The large molecular overlap is a signature of good electrical properties, as suggested by theoretical (Coropceanu *et al.*, 2007) and experimental studies (Mei *et al.*, 2013).

### S2. Experimental

Dibenzotetrathiafulvalene (DBTTF) and pyromellitic dianhydride (PMDA), both obtained from Sigma Aldrich, were separately dissolved in xylenes and acetonitrile, respectively. The solid weights of the compounds were measured in the molar ratio 1:1. The solution concentrations were saturated, such that all of parent compound dissolved in as little solvent as possible. The solutions were mixed, and the complex was then crystallized by slow evaporation under ambient conditions. After about two days of evaporation, crystals were obtained as green-gold plates with approximate dimensions of 0.20 mm *x* 0.20 mm *x* 0.02 mm.

### S3. Refinement

The hydrogen atoms were included in the structural model as fixed atoms (using idealized *sp*^2^-hybridized geometry and C—H bond lengths of 0.94 Å) "riding" on their respective carbon atoms. The isotropic thermal parameter of each hydrogen atom was fixed at a value 1.2 times the equivalent isotropic thermal parameter of the carbon atom to which it is covalently bonded.

### Crystal data

**Table:**

C_10_H_2_O_6_·C_14_H_8_S_4_	*Z* = 1
*M**_r_* = 522.56	*F*(000) = 266
Triclinic, *P*1	*D*_x_ = 1.658 Mg m^−^^3^
Hall symbol: -P 1	Mo *K*α radiation, λ = 0.71073 Å
*a* = 7.2292 (4) Å	Cell parameters from 5268 reflections
*b* = 8.9572 (5) Å	θ = 3.5–31.3°
*c* = 9.5224 (5) Å	µ = 0.50 mm^−^^1^
α = 70.051 (1)°	*T* = 213 K
β = 68.712 (1)°	Plate, green-gold
γ = 70.136 (1)°	0.20 × 0.20 × 0.02 mm
*V* = 523.39 (5) Å^3^	

### Data collection

**Table:**

Bruker APEX CCD diffractometer	3023 independent reflections
Radiation source: sealed x-ray tube	2668 reflections with *I* > 2σ(*I*)
Graphite monochromator	*R*_int_ = 0.021
φ and ω scans	θ_max_ = 30.0°, θ_min_ = 3.5°
Absorption correction: multi-scan (*SADABS*; Sheldrick, 2012)	*h* = −10→10
*T*_min_ = 0.703, *T*_max_ = 0.746	*k* = −12→12
10004 measured reflections	*l* = −13→13

### Refinement

**Table:**

Refinement on *F*^2^	Primary atom site location: structure-invariant direct methods
Least-squares matrix: full	Hydrogen site location: inferred from neighbouring sites
*R*[*F*^2^ > 2σ(*F*^2^)] = 0.032	H-atom parameters constrained
*wR*(*F*^2^) = 0.091	*w* = 1/[σ^2^(*F*_o_^2^) + (0.0529*P*)^2^ + 0.115*P*] where *P* = (*F*_o_^2^ + 2*F*_c_^2^)/3
*S* = 1.07	(Δ/σ)_max_ < 0.001
3023 reflections	Δρ_max_ = 0.30 e Å^−^^3^
154 parameters	Δρ_min_ = −0.33 e Å^−^^3^
0 restraints	

### Special details

**Table:**

Experimental. Absorption correction: data were corrected for scaling and absorption effects using the multi-scan technique [SADABS (Sheldrick, 2012)]. The ratio of minimum to maximum apparent transmission was 0.942.
Geometry. All e.s.d.'s (except the e.s.d. in the dihedral angle between two l.s. planes) are estimated using the full covariance matrix. The cell e.s.d.'s are taken into account individually in the estimation of e.s.d.'s in distances, angles and torsion angles; correlations between e.s.d.'s in cell parameters are only used when they are defined by crystal symmetry. An approximate (isotropic) treatment of cell e.s.d.'s is used for estimating e.s.d.'s involving l.s. planes.

### Fractional atomic coordinates and isotropic or equivalent isotropic displacement parameters (Å^2^)

**Table:**

	*x*	*y*	*z*	*U*_iso_*/*U*_eq_	
S1	−0.06704 (4)	0.15387 (4)	0.15691 (4)	0.02936 (10)	
S2	−0.31329 (5)	−0.02880 (4)	0.11851 (4)	0.03025 (10)	
C1	−0.08003 (17)	0.02644 (15)	0.05769 (14)	0.0250 (2)	
C2	−0.32321 (18)	0.17849 (14)	0.27098 (14)	0.0245 (2)	
C3	−0.43823 (18)	0.09185 (14)	0.25298 (14)	0.0246 (2)	
C4	−0.64247 (19)	0.10259 (16)	0.34069 (16)	0.0291 (3)	
H4	−0.7200	0.0443	0.3290	0.035*	
C5	−0.7294 (2)	0.20070 (17)	0.44543 (16)	0.0333 (3)	
H5	−0.8672	0.2096	0.5042	0.040*	
C6	−0.6146 (2)	0.28587 (17)	0.46427 (15)	0.0332 (3)	
H6	−0.6754	0.3511	0.5362	0.040*	
C7	−0.4108 (2)	0.27550 (16)	0.37773 (15)	0.0292 (3)	
H7	−0.3334	0.3329	0.3909	0.035*	
O1	0.08852 (14)	0.63648 (12)	0.27821 (12)	0.0338 (2)	
O2	0.30802 (18)	0.75698 (14)	0.29107 (13)	0.0441 (3)	
O3	−0.05194 (14)	0.48790 (13)	0.21480 (13)	0.0395 (2)	
C8	0.2784 (2)	0.67350 (16)	0.23163 (15)	0.0301 (3)	
C9	0.41355 (18)	0.59058 (14)	0.10654 (14)	0.0237 (2)	
C10	0.30252 (17)	0.50435 (14)	0.08434 (14)	0.0237 (2)	
C11	0.09415 (18)	0.53456 (16)	0.19353 (15)	0.0282 (2)	
C12	0.61565 (17)	0.58997 (14)	0.02281 (14)	0.0252 (2)	
H12	0.6905	0.6484	0.0375	0.030*	

### Atomic displacement parameters (Å^2^)

**Table:**

	*U*^11^	*U*^22^	*U*^33^	*U*^12^	*U*^13^	*U*^23^
S1	0.02281 (15)	0.03348 (17)	0.03676 (18)	−0.00701 (12)	−0.00525 (12)	−0.01796 (14)
S2	0.02316 (15)	0.03528 (18)	0.03745 (19)	−0.00885 (12)	−0.00331 (12)	−0.01913 (14)
C1	0.0214 (5)	0.0260 (5)	0.0281 (6)	−0.0048 (4)	−0.0051 (4)	−0.0102 (5)
C2	0.0249 (5)	0.0227 (5)	0.0237 (5)	−0.0048 (4)	−0.0055 (4)	−0.0057 (4)
C3	0.0234 (5)	0.0239 (5)	0.0246 (5)	−0.0043 (4)	−0.0059 (4)	−0.0062 (4)
C4	0.0249 (5)	0.0291 (6)	0.0303 (6)	−0.0072 (5)	−0.0050 (5)	−0.0060 (5)
C5	0.0282 (6)	0.0341 (7)	0.0274 (6)	−0.0061 (5)	0.0008 (5)	−0.0063 (5)
C6	0.0378 (7)	0.0298 (6)	0.0255 (6)	−0.0067 (5)	−0.0007 (5)	−0.0094 (5)
C7	0.0347 (6)	0.0266 (6)	0.0261 (6)	−0.0084 (5)	−0.0059 (5)	−0.0083 (5)
O1	0.0265 (4)	0.0378 (5)	0.0343 (5)	−0.0065 (4)	0.0015 (4)	−0.0179 (4)
O2	0.0466 (6)	0.0508 (6)	0.0436 (6)	−0.0154 (5)	−0.0036 (5)	−0.0288 (5)
O3	0.0246 (5)	0.0465 (6)	0.0462 (6)	−0.0133 (4)	−0.0009 (4)	−0.0153 (5)
C8	0.0295 (6)	0.0318 (6)	0.0280 (6)	−0.0075 (5)	−0.0028 (5)	−0.0119 (5)
C9	0.0242 (5)	0.0249 (5)	0.0228 (5)	−0.0061 (4)	−0.0051 (4)	−0.0082 (4)
C10	0.0204 (5)	0.0253 (5)	0.0239 (5)	−0.0065 (4)	−0.0048 (4)	−0.0050 (4)
C11	0.0234 (5)	0.0288 (6)	0.0287 (6)	−0.0054 (4)	−0.0031 (4)	−0.0084 (5)
C12	0.0239 (5)	0.0270 (6)	0.0274 (6)	−0.0082 (4)	−0.0065 (4)	−0.0086 (5)

### Geometric parameters (Å, º)

**Table:**

S1—C1	1.7543 (13)	C6—H6	0.9400
S1—C2	1.7560 (12)	C7—H7	0.9400
S2—C3	1.7505 (13)	O1—C11	1.3923 (16)
S2—C1	1.7567 (12)	O1—C8	1.3993 (16)
C1—C1^i^	1.353 (2)	O2—C8	1.1878 (17)
C2—C7	1.3967 (17)	O3—C11	1.1907 (16)
C2—C3	1.3985 (17)	C8—C9	1.4800 (17)
C3—C4	1.3963 (16)	C9—C12	1.3864 (16)
C4—C5	1.3892 (19)	C9—C10	1.3933 (16)
C4—H4	0.9400	C10—C12^ii^	1.3839 (16)
C5—C6	1.391 (2)	C10—C11	1.4836 (16)
C5—H5	0.9400	C12—C10^ii^	1.3838 (16)
C6—C7	1.3913 (19)	C12—H12	0.9400
			
C1—S1—C2	95.38 (6)	C6—C7—C2	118.93 (13)
C3—S2—C1	95.34 (6)	C6—C7—H7	120.5
C1^i^—C1—S1	122.00 (13)	C2—C7—H7	120.5
C1^i^—C1—S2	122.26 (13)	C11—O1—C8	110.17 (10)
S1—C1—S2	115.74 (6)	O2—C8—O1	121.25 (13)
C7—C2—C3	120.37 (11)	O2—C8—C9	131.52 (13)
C7—C2—S1	123.12 (10)	O1—C8—C9	107.23 (11)
C3—C2—S1	116.50 (9)	C12—C9—C10	122.97 (11)
C4—C3—C2	120.30 (12)	C12—C9—C8	129.32 (11)
C4—C3—S2	122.83 (10)	C10—C9—C8	107.70 (10)
C2—C3—S2	116.88 (9)	C12^ii^—C10—C9	123.01 (10)
C5—C4—C3	119.04 (12)	C12^ii^—C10—C11	129.51 (11)
C5—C4—H4	120.5	C9—C10—C11	107.48 (11)
C3—C4—H4	120.5	O3—C11—O1	121.29 (12)
C4—C5—C6	120.69 (12)	O3—C11—C10	131.31 (13)
C4—C5—H5	119.7	O1—C11—C10	107.40 (10)
C6—C5—H5	119.7	C10^ii^—C12—C9	114.03 (10)
C5—C6—C7	120.66 (13)	C10^ii^—C12—H12	123.0
C5—C6—H6	119.7	C9—C12—H12	123.0
C7—C6—H6	119.7		
			
C2—S1—C1—C1^i^	176.20 (15)	C11—O1—C8—O2	−178.78 (13)
C2—S1—C1—S2	−4.14 (8)	C11—O1—C8—C9	0.82 (14)
C3—S2—C1—C1^i^	−176.37 (15)	O2—C8—C9—C12	−0.6 (2)
C3—S2—C1—S1	3.97 (8)	O1—C8—C9—C12	179.86 (12)
C1—S1—C2—C7	−178.74 (11)	O2—C8—C9—C10	178.37 (15)
C1—S1—C2—C3	2.71 (10)	O1—C8—C9—C10	−1.17 (14)
C7—C2—C3—C4	0.54 (18)	C12—C9—C10—C12^ii^	0.2 (2)
S1—C2—C3—C4	179.13 (9)	C8—C9—C10—C12^ii^	−178.82 (11)
C7—C2—C3—S2	−179.00 (9)	C12—C9—C10—C11	−179.90 (11)
S1—C2—C3—S2	−0.41 (13)	C8—C9—C10—C11	1.05 (13)
C1—S2—C3—C4	178.35 (11)	C8—O1—C11—O3	179.96 (12)
C1—S2—C3—C2	−2.12 (10)	C8—O1—C11—C10	−0.18 (14)
C2—C3—C4—C5	0.16 (18)	C12^ii^—C10—C11—O3	−0.9 (2)
S2—C3—C4—C5	179.68 (10)	C9—C10—C11—O3	179.27 (14)
C3—C4—C5—C6	−0.7 (2)	C12^ii^—C10—C11—O1	179.29 (12)
C4—C5—C6—C7	0.5 (2)	C9—C10—C11—O1	−0.57 (13)
C5—C6—C7—C2	0.2 (2)	C10—C9—C12—C10^ii^	−0.21 (19)
C3—C2—C7—C6	−0.73 (18)	C8—C9—C12—C10^ii^	178.62 (12)
S1—C2—C7—C6	−179.23 (10)		

Symmetry codes: (i) −*x*, −*y*, −*z*; (ii) −*x*+1, −*y*+1, −*z*.
